# Immune Modulation by Vitamin D and Its Relevance to Food Allergy

**DOI:** 10.3390/nu7085271

**Published:** 2015-07-27

**Authors:** Noor H. A. Suaini, Yuxia Zhang, Peter J. Vuillermin, Katrina J. Allen, Leonard C. Harrison

**Affiliations:** 1 Murdoch Childrens Research Institute, The Royal Children’s Hospital, Parkville, VIC 3052, Australia; E-Mails: noor.suaini@mcri.edu.au (N.H.A.S.); peter.vuillermin@deakin.edu.au (P.J.V.); 2 Department of Paediatrics, University of Melbourne, Parkville, VIC 3052, Australia; 3 The Walter and Eliza Hall Institute of Medical Research, Parkville VIC 3052, Australia; E-Mails: yzhang@wehi.edu.au (Y.Z.); harrison@wehi.edu.au (L.C.H.); 4 Department of Medical Biology, University of Melbourne, Parkville, VIC 3052, Australia; 5 Child Health Research Unit, Barwon Health, Geelong, VIC 3220, Australia; 6 Deakin University, Geelong, VIC 3220, Australia; 7 Department of Allergy and Immunology, The Royal Children’s Hospital, Parkville, VIC 3052, Australia; 8 University of Manchester, Oxford Road, Manchester M13 9PL, UK

**Keywords:** vitamin D, innate immunity, adaptive immunity, food allergy, 1,25(OH)_2_D_3_, T cells, inflammation, metabolism, deficiency

## Abstract

Apart from its classical function in bone and calcium metabolism, vitamin D is also involved in immune regulation and has been linked to various cancers, immune disorders and allergic diseases. Within the innate and adaptive immune systems, the vitamin D receptor and enzymes in monocytes, dendritic cells, epithelial cells, T lymphocytes and B lymphocytes mediate the immune modulatory actions of vitamin D. Vitamin D insufficiency/deficiency early in life has been identified as one of the risk factors for food allergy. Several studies have observed an association between increasing latitude and food allergy prevalence, plausibly linked to lower ultraviolet radiation (UVR) exposure and vitamin D synthesis in the skin. Along with mounting epidemiological evidence of a link between vitamin D status and food allergy, mice and human studies have shed light on the modulatory properties of vitamin D on the innate and adaptive immune systems. This review will summarize the literature on the metabolism and immune modulatory properties of vitamin D, with particular reference to food allergy.

## 1. Introduction

Classically notable for its effects on calcium metabolism and bone mineralisation, vitamin D is now recognised to have protean effects, including on the immune system. The immunological significance of vitamin D was first recognised when the vitamin D receptor (VDR) was identified in lymphocytes [1,2]. Vitamin D deficiency has been associated with various immune diseases, including allergic [3,4] and autoimmune diseases [5,6]. In particular, an increase in the population prevalence of vitamin D insufficiency has been observed in parallel with an increase in food allergy [7]. Food allergy has been associated with indicators of vitamin D insufficiency, in particular vitamin D synthesis in the skin and increasing latitude, which may be due to lower UV exposure [8,9]. Additionally, findings from our HealthNuts study, a population-based study of food allergy in Melbourne infants, suggest a potential role of vitamin D insufficiency at 12 months of age in the development of food allergy [7]. However, the biological mechanisms underlying these epidemiological associations remain unclear, and some research has suggested that vitamin D excess may also increase the risk of abnormal immune responses and an increased risk of food allergy [10].

The VDR regulates the actions of immune cells, such as monocytes, dendritic cells (DCs), T and B cells, in an interplay between the innate and adaptive immune systems [11,12,13,14]. As the first barrier of defence, the innate immune system protects against invading microorganisms [15]. Several mechanisms have been postulated for the effects of vitamin D on the innate and adaptive immune system—to reduce inflammation, promote immune tolerance and enhance gut epithelial integrity—but the mechanisms by which vitamin D may influence the risk of food allergy are not clear. In this review, the metabolism of vitamin D and its immune modulatory properties in relation to food allergy will be summarised. 

## 2. Regulation and Metabolism of 1,25(OH)_2_D

Vitamin D is a fat-soluble vitamin that can be acquired through dietary sources, although most is produced in the skin following sunlight exposure. Vitamin D is the general term encompassing both vitamin D_2_ and D_3_. Vitamin D_2_, also known as ergocalciferol, is produced when ultraviolet light, in particular the UVB band of sunlight, acts on ergosterol, which is found mainly in yeast and plants [16,17]. On the other hand, vitamin D_3_, cholecalciferol, is formed when 7-dehydrocholesterol found in the skin absorbs the UVB radiation [16,18]. Both vitamin D_2_ and D_3_ generally undergo the same processes to form calcitriol (1,25(OH)_2_D). However, due to differences in their chemical structures, there are minor differences in the types of metabolites produced and the sites of hydroxylation [19]. Studies have shown that vitamin D binding protein (VDBP) has a higher affinity for vitamin D_3_ than D_2_ [20]. This results in vitamin D_3_ having a longer half-life and being more potent than D_2_ [21,22,23]. In this section, the metabolic processes will be generalised to include both forms of vitamin D.

### 2.1. Renal Synthesis of 1,25(OH)_2_D

**Figure 1:**
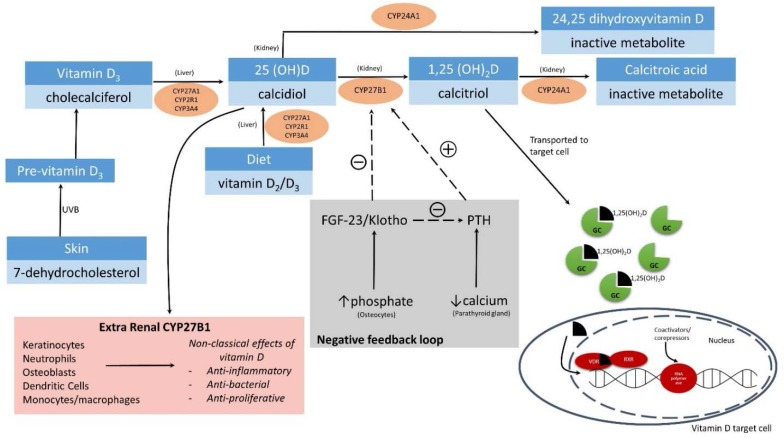
Vitamin D metabolism and associated enzymes. Vitamin D from the diet and skin undergoes several hydroxylation steps to produce the biologically-active form of vitamin D, 1,25(OH)_2_D. In circulation, 1,25(OH)_2_D bound to the vitamin D binding protein (encoded by *GC* gene) is transported to the vitamin D target cells. Once it reaches the target cell, it dissociates from the binding protein and translocates to the nucleus, where it binds to the vitamin D receptor (VDR) and heterodimerizes with the retinoid X receptor (RXR). Recruitment of transcription factors results in the activation or repression of gene transcription. 1,25(OH)_2_D synthesis is regulated by feedback mechanisms involving fibroblast growth factor 23 (FGF-23) and parathyroid hormone (PTH). In the parathyroid gland, low serum calcium levels induce the secretion of PTH and activation of CYP27B1, resulting in the production of 1,25(OH)_2_D. As a second feedback loop, a high serum phosphate level triggers the secretion of FGF-23 from osteocytes and inhibits CYP27B1 synthesis. Activation of CYP24A1 also converts the bioactive vitamin D_3_ into inactive metabolites for secretion in bile.

Sterol 27-hydroxylase (CYP27A1) and vitamin D 25-hydroxylase (CYP2R1), two cytochromes expressed in the liver, hydroxylate vitamin D to 25-hydroxyvitamin D (25(OH)D) [24]. Other cytochrome P450 vitamin D 25 hydroxylases, such as CYP3A4 [25,26,27] and CYP2J2 [28], have also been shown to have some 25 hydroxylase activity. In the kidney, 25(OH)D then undergoes further hydroxylation by 25-hydroxyvitamin D-1α-hydroxylase (CYP27B1) to produce the biologically-active form of vitamin D, 1,25-dihydroxyvitamin D (1,25(OH)_2_D) (Figure 1). Vitamin D and its plasma metabolites are mostly transported by VDBP (encoded by the *GC* gene), with a smaller percentage bound to albumin [29]. On reaching the target cells, 1,25(OH)_2_D dissociates from the VDBP, diffuses into the cell and binds to the nuclear VDR to initiate gene transcription [30,31,32,33]. It is worth noting that the VDBPs do not actually facilitate 1,25(OH)_2_D entry into the cell [34]. Finally, 25(OH)D and 1,25(OH)_2_D may be metabolically inactivated through hydroxylation by 24-hydroxylase (CYP24A1), hence limiting its availability [35,36].

Renal production of 1,25(OH)_2_D is tightly regulated by a feedback loop at the cellular level, primarily through the actions of parathyroid hormone (PTH) and fibroblast growth factor 23 (FGF-23) (Figure 1). In response to low circulating calcium levels, PTH is secreted by the parathyroid gland to stimulate CYP27B1 production by primary renal tubules [36,37,38]. As renal production of 1,25(OH)_2_D increases, VDR binds to *CYP27B1* promoter to repress its expression and, thereby, production of 1,25(OH)_2_D. 1,25(OH)_2_D increases the uptake of calcium and inhibits production and secretion of PTH [38]. 

While PTH is essential in maintaining blood calcium levels, FGF-23 plays a role in mineral homeostasis determined by genes regulating serum phosphate and vitamin D metabolism. Increased serum phosphate induces a marked increase in *FGF-23* expression and FGF-23 secretion by bone cells [39]. Concurrently, FGF-23 action reduces renal expression of *CYP27B1*, leading to decreased serum 1,25(OH)_2_D [40]. FGF-23 negatively regulates CYP27B1 activity together with the transmembrane protein Klotho, which acts as a co-receptor essential for the activation of FGF signaling by FGF-23 [38,39,40]. Renal 1,25(OH)_2_D concentrations are thus tightly regulated by a network of feedback loops, which includes the inhibition of CYP27B1 by FGF-23/Klotho, activation of CYP27B1 by low circulating calcium and increased PTH secretion and activation of CYP24A1 to initiate degradation of metabolites [12,32,37,41,42].

### 2.2. Extra-Renal Synthesis of 1,25(OH)_2_D

Extra-renal synthesis of 1,25(OH)_2_D is present in many tissues, such as parathyroid glands, keratinocytes and immune cells [37,38]. For example, CYP27B1 is expressed in T cells [43], activated macrophages and DCs [43,44,45], allowing the formation of 1,25(OH)_2_D in the immune cells. Unlike in the kidney, regulation of extra-renal CYP27B1 is generally under the control of immune stimuli and not by the classical feedback loop involving PTH and FGF-23 [13,38,46]. In macrophages, CYP27B1 is induced after Toll-like receptor (TLR) ligation or stimulation by interferon-γ (IFN-γ) [37,45,47]. *CYP24A1* is transcribed as an enzymatically-inactive splice variant, which prevents the breakdown of 25(OH)D and 1,25(OH)_2_D [48,49]. As a result, overexpression of unregulated CYP27B1 in macrophages may potentially lead to excessive 1,25(OH)_2_D production, contributing to pathological diseases, such as sarcoidosis [49,50]. Sites of extra-hepatic 25-hydroxylase activity have also been reported [51], with the detection of *CYP27* mRNA in the bone [52] and white blood cells [53,54]. Novel extra-hepatic P450 enzymes, such as CYP2S1 and CYP2U1, have also been identified [55]. 

### 2.3. Genomic and Non-Genomic (Rapid) Signaling

Binding of 1,25(OH)_2_D to the VDR occurs with high affinity and selectivity, preventing the precursor, 25(OH)D, from activating the VDR under normal circumstances [56]. The VDR-1,25(OH)_2_D complex heterodimerizes with retinoid X receptor (RXR) to bind to the vitamin D response element (VDRE) located in the promoter region of vitamin D-responsive genes. This leads to recruitment of co-activators (e.g., SRC1, CBP, MED1) or co-repressors (e.g., NcoR, SMRT) to regulate transcription of 1,25(OH)_2_D-responsive genes [57]. However, the VDR may also regulate gene expression in a 1,25(OH)_2_D-independent manner via recruitment of gene-specific co-regulatory complexes [56]. The VDR can be post-translationally modified by phosphorylation, although the functional significance of this is uncertain [58,59]. 

While the genomic signaling is reliant on responses to the nuclear VDR, non-genomic signaling utilises different signal transduction pathways [60]. It was demonstrated that rapid signaling is mediated through VDRs associated within caveolae or lipid rafts on the plasma membrane of certain cells [61]. Examples of systems that involve rapid signaling include intestinal calcium transport in a vitamin D-replete chick [60] and 1,25(OH)_2_D_3_ modulation of osteoblast ion channel responses [62]. Non-genomic signaling pathways triggered by 1,25(OH)_2_D_3_ may be mediated through activation of second messengers, such as protein kinase C (PKC) [63], intracellular increase in calcium and modulation of phospholipase C and adenylate cyclase [64]. In particular, 1,25(OH)_2_D_3_ has been shown to directly activate PKC at physiological concentrations, with PKC acting as a membrane-associated receptor for the hormone [65]. Two isoforms of PKC, PKCβI and PKCζ, have been shown to be involved in 1,25(OH)_2_D_3_ induction of rat cytochrome P450C24 (CYP24) expression, while the other PKC enzymes, PKCα, PKCδ and PKCε, were not essential [66,67]. Using human embryonic kidney 293T cells, Nutchey *et al.* [66] found that JNK activity, but not extracellular signal-regulated kinase (ERK) 1/2, was required for 1,25(OH)_2_D_3_ to induce the expression of *CYP24* gene. However, the opposite was observed in monkey kidney fibroblast COS-1 cells, in which JNK was not found to mediate CYP24, but instead, either ERK1/ERK2 or ERK5 modules or both were required [68]. Given that CYP24 null mice exhibited elevated 1,25(OH)_2_D_3_ levels due to impaired catabolism, regulation of CYP24 expression may prevent 1,25(OH)_2_D_3_-induced toxicity [69,70,71].

### 2.4. Definitions of Vitamin D Status

25(OH)D_3_ has a half-life of approximately 15 days as compared to only 15 h for 1,25(OH)_2_D_3_ [72]. Thus, serum 25(OH)D_3_ circulates at a much higher concentration and is typically used as a marker of vitamin D status. Australian guidelines stipulated that mild vitamin D deficiency is defined as a serum 25(OH)D concentration of 30–49 nmol/L, moderate deficiency as 12.5–29 nmol/L and severe vitamin D deficiency as <12.5 nmol/L [73]. The optimal level of serum 25(OH)D is unknown, although concentrations of ≥50 nmol/L are generally considered adequate [17,74]. Several studies found a positive correlation between 1,25(OH)_2_D_3_ and 25(OH)D_3_ [75,76,77]. Nordin *et al.* [76] suggested that the relationship between the two metabolites is biphasic; positive when 25(OH)D_3_ is normal and negative when 25(OH)D_3_ is below the normal range. However, findings from some earlier studies suggested that serum concentrations of the two vitamin D metabolites are not correlated and attributed findings of a positive relationship to methodological errors [78,79]. 

## 3. Regulation of Immune Function by Vitamin D

### 3.1. Innate Immunity

The innate immune system, as the first barrier of immune defence, guards against invading foreign microorganisms and contributes to the maintenance of immune homeostasis [15]. Foreign microorganisms express pathogen-associated molecular patterns (PAMPs) that are recognised by pattern recognition receptors, such as TLRs expressed by the innate immune cells [80]. Depending on which TLRs are activated by PAMPs, the innate immune system will then mediate responses, such as the production of antimicrobial peptides and cytokines and the apoptosis of host cells [80]. Vitamin D has been found to induce antimicrobial peptide synthesis in the innate immune cells, to dampen excessive inflammation and to inhibit the production of pro-inflammatory cytokine [81,82]. In preventing infection, vitamin D induces antimicrobial peptide synthesis [83,84] through VDREs present in the promoter regions of genes encoding antimicrobial peptides *cathelicidin* (*hCAP18*) and β*-defensin* (*DEFB*) [85]. Production of cathelicidin, which is upregulated in the presence of 1,25(OH)_2_D_3_ [85], is essential for antimicrobial defences and the cytotoxic activity of natural killer cells against tumour cells [82,86]. Additionally, cathelicidin has also been shown to mediate vitamin D-induced autophagy in monocytes/macrophages [87,88]. Yuk *et al.* [88] demonstrated that 1,25(OH)_2_D_3_ is required for co-localization of mycobacterial phagosomes with autophagosomes. Another vitamin D-related mechanism postulated to prevent infection is enhancement of nitric oxide production by macrophages and expression of inducible nitric oxide synthase [89]. Although this has been shown in mouse models, its significance in humans is still questionable, as the amount of nitric oxide produced *in vitro* by human macrophages was negligible [90]. 

Complementing its antimicrobial properties, 1,25(OH)_2_D_3_ acts to suppress excessive inflammation. 1,25(OH)_2_D_3_ represses the expression of TLRs [45,84] and inhibits the production of pro-inflammatory cytokines by DCs and innate immune cells [91,92]. VDR activation inhibits DC differentiation and maturation, which is reflected by decreased surface expression of MHC class II, co-stimulatory molecules (CD40, CD80 and CD86) and other maturation-induced surface markers, such as CD83 [93,94,95,96]. As immature DCs are ‘tolerogenic’, inhibition of DC differentiation by 1,25(OH)_2_D_3_ contributes to T cell tolerance and adaptive immune homeostasis (discussed below). 

Consistent with its anti-inflammatory role, 1,25(OH)_2_D_3_ may also inhibit type 1 T-helper (Th1) cell responses by suppression of IL-12 production by DCs [94,97]. This is due to disruption by the 1,25(OH)_2_D_3_-VDR complex of NF-κB binding to IL-12 promoter regions and, hence, inhibition of IL-12 mRNA expression [95,98]. While IL-12 synthesis is suppressed, IL-10 production by DCs is enhanced, together with induction of type 1 (IL-10 secreting) regulatory cells (Tr1) [94,99] and a shift from a Th1 to type 2 T-helper (Th2) phenotype [100]. Enhanced IL-10 production has also been observed in mouse mast cells *in vitro* in the presence of 1,25(OH)_2_D_3_ [101]. Augmented IL-10 synthesis contributing to a suppressed mast cell-mediated inflammation, may dampen IgE-dependent allergic reactions [102]. Clinically, the beneficial effects of vitamin D were observed in a recent clinical trial that showed the resolution of inflammatory responses in 95 participants treated with adjunctive vitamin D supplementation in addition to standard tuberculous therapy [103]. 

### 3.2. Adaptive Immunity

Signals from innate immune cells determine the fate of differentiating T cells and B cells [104,105]. Hewison [106] suggested four plausible mechanisms through which serum 25(OH)D may modulate T cell function: (i) direct effects mediated via systemic 1,25(OH)_2_D; (ii) indirect effects via antigen presentation by monocytes and macrophages to T cells, mediated by DC expression of CYP27B1 and intracrine synthesis of 1,25(OH)_2_D; (iii) direct effects of 1,25(OH)_2_D on T cells following the synthesis of the biologically-active vitamin D by CYP27B1-expressing monocytes or DCs (paracrine mechanism); and (iv) intracrine conversion of 25(OH)D to 1,25(OH)_2_D by T cells. 

A group of T cells that may also be activated by 1,25(OH)_2_D are regulatory T cells (Tregs). Tregs suppress other immune cells and contribute to the maintenance of immune homeostasis by a range of mechanisms, including cell to cell contact and secretion of anti-inflammatory factors, such as IL-10 and TGF-β [107,108,109]. They can be classified into two major subtypes: those that are naturally occurring in the thymus and those derived from peripheral CD4^+^ T cells following antigen stimulation [110,111]. Peripheral differentiation of Tregs is induced when 1,25(OH)_2_D_3_ represses the differentiation and maturation of DCs to generate tolerogenic immature DCs [42,106]. 1,25(OH)_2_D_3_ can also bind to the retinoic acid receptor to promote peripheral Treg differentiation from naive CD4^+^ T cells [112] into IL-10-secreting-Tr1 Tregs [11]. This suppressive activity may also be induced by VDR agonists [113]. In a clinical trial of 46 individuals, 140,000 IU of vitamin D supplementation were associated with an increased Treg frequency in peripheral circulation after four weeks [114].

Studies have also shown that T cell proliferation and function is inhibited in response to 1,25(OH)_2_D_3_ (calcitriol) [94,97,115,116] by a reduction in IL-2 production [112]. T cells treated directly with calcitriol or its analogues had decreased expression (in the absence of antigen-presenting cells) of Th1 (IL-2, TNF-α, IFN-γ), Th9 (IL-9) and Th22 (IL-22) cytokines [97,117,118], but increased production of anti-inflammatory Th2 cytokines (IL-3, IL-4, IL-5, IL-10) [119]. While the immunosuppressive effects of vitamin D on Th1 cells are clear, the effects of vitamin D on Th2 cytokine expression are contradictory and yet to be clarified. Some studies showed that 1,25(OH)_2_D_3_ favours Th2 cells by upregulating the expression of Th2-specific transcription factors GATA-3 and c-Maf, as well as IL-4 in mice [119,120]. Others contradicted these findings, with c-Maf being undetectable in the presence of vitamin D [121] and decreased Th2 cytokine levels [112,122]. Apart from T cells, evidence suggest that IgE production by B cells is also suppressed by 1,25(OH)_2_D_3_ [123].

## 4. The Role of Vitamin D Status in Failed Oral Tolerance and Development of Food Allergy

IgE-mediated food allergy, the most common form of food allergy [124], usually develops in the first year of life, presumably as a result of aberrant immune development. Food allergy is generally Th2-biased and is characterised by the secretion of IL-4, IL-5, IL-9 and IL-13 and allergen-specific IgE antibody [125,126]. Early IgE production to food allergens (sensitization) occurs through oral and gut mucosal or cutaneous food exposures [126,127,128]. Allergen-specific IgE antibodies bind to high-affinity Fcε receptors (FcεRI) on the surface of mast cells and basophils upon re-exposure to the food constituents [129]. This triggers degranulation of mast cells and basophils, releasing the preformed mediators, histamine, tryptase and TNFs, which promote oedema, erythema and itch [124,126,130]. Newly-synthesized mediators, such as leukotrienes (e.g., LTC4), prostaglandins (e.g., PGD_2_) and cytokines (e.g., IL-3, IL-5), lead to the recruitment of inflammatory cells responsible for IgE-mediated late-phase responses [124,131]. Class-switching to pro-allergic IgE antibody production is promoted by T cell production of IL-4 and IL-13 [132]. While sensitization predisposes individuals to food allergy, they may not develop the clinical manifestations mentioned above [133]. Currently, it is not well understood why certain individuals do not develop food allergy despite producing IgE antibodies.

Controversy remains regarding the role of vitamin D in the development of food allergy. On the one hand, some research suggests that vitamin D insufficiency increases the risk of IgE-mediated food allergy [7] and food sensitization [134]. By contrast, vitamin D excess in pregnancy and at birth has also been associated with an increased risk of food allergy [135]. It is thought that the lower number of Tregs associated with high cord blood vitamin D may compromise immune tolerance, since a low Treg count has been previously found to predict the development of early atopic dermatitis [136]. The contribution of vitamin D in allergic diseases is evident in mouse studies [137,138]. Production of thymic stromal lymphopoietin (TSLP) in keratinocytes is essential in promoting allergic sensitization through an impaired skin barrier and eventual development of allergic asthma [137]. The application of vitamin D or an analogue induces *TSLP* transcription by enhancing its promoter activity [138]. Although a unifying hypothesis for the role of vitamin D in food allergy development would suggest a U-shaped relationship [10,139], the evidence remains thin and as yet to be validated by randomized controlled trials.

Evidence for the role of low vitamin D in food allergy development is currently the strongest. Vassallo *et al.* [140] proposed a “multiple-hit model” where low vitamin D results in an increased susceptibility to gastrointestinal infections and compromised barrier defences. In the presence of an altered microbial ecology of the gastrointestinal tract and a lowered immune tolerance, this may predispose an individual to allergic responses to food antigens. This is supported by emerging research showing the effects of 1,25(OH)_2_D_3_ to promote mucosal barrier function [141], inhibit pro-allergic immune responses and promote immunologic tolerance [14]. In maintaining mucosal barrier function, 1,25(OH)_2_D_3_ enhances the expression of genes encoding proteins required for epithelial tight junctions (e.g., occludin), gap junctions (e.g., connexion 43) and adherens junctions (e.g., E-cadherin) [142,143,144,145]. 

The vitamin D mechanisms contributing to immune tolerance include the induction of tolerogenic DCs [146] and Tregs [14] and inhibition of IgE production in B cells [147]. Repeated exposures to low doses of food antigen have been found to be optimal for the development of Tregs [108], for oral tolerance. This may in part be established by mucosal tolerogenic DCs to induce Tregs in the mesenteric lymph nodes [148]. Mice studies showed that Tregs were able to alleviate clinical signs of immediate-type hypersensitivity reactions in IgE-mediated food allergy [149,150]. Studies in human subjects have also shown that DCs of allergic patients are less responsive to Th1-inducing stimuli, hence increasing susceptibility to a Th2-skewed profile [151].

Suppression of IgE production is also modulated by anti-CD40 antibody- and IL-4-stimulated B cells exposed to the vitamin D analogue, EB1089 [152], and this finding was replicated *in vivo* in an allergy mouse model [153]. Human B cell experiments have also shown that 1,25(OH)_2_D_3_ suppresses IgE antibody class switch partly through inhibition of NF-κB and epsilon germ-line transcription (εGLT) [152]. IL-10 production is however inhibited in anti-CD40/IL-4-activated B cells, either directly, through binding of VDR to the promoter region of IL-10, and/or indirectly by the modulation of calcium [147]. 

Some epidemiological evidence suggests that vitamin D excess may also contribute to food allergy pathogenesis. The relationship between 25(OH)D concentrations and allergy may not be linear, with one study proposing a U-shaped association between vitamin D_3_ with total serum IgE concentration production in adults [10]. In this study, elevated IgE levels were observed in both groups of participants with the lowest (<25 nmol/L) and highest (>135 nmol/L) concentrations of serum 25(OH)D_3_. This dual effect of vitamin D is supported by mouse data, which showed that while 1,25(OH)_2_D_3_ downregulates airway eosinophilia through reduction in inflammation, it also enhances allergen-specific T cell activation, systemic IgE levels and IL-4 and IL-13 secretion [154]. 

Similarly, cord blood concentrations of 25(OH)D also had a U-shaped association with aeroallergen-specific IgE, with an odds ratio of 2.4 and four for low and high concentrations of 25(OH)D, respectively [155]. In measuring cord blood 25(OH)D_3_, a recent study suggested a strong correlation between maternal vitamin D level and cord blood levels of neonates [156], and this is dependent on the ability of 25(OH)D_3_ to cross the placenta [157]. As such, the role of maternal vitamin D status and vitamin D immunoregulation *in utero* also warrants further investigation.

## 5. Conclusions

There is significant interest in the role of vitamin D for optimal immune health. Recent epidemiological evidence suggests that both insufficiency and excess of vitamin D may contribute to the failure of oral tolerance and subsequent food allergy in infants. It is evident that vitamin D has wide-ranging effects on the immune system, but how it modulates immune function in food allergy is not clear. While food allergy is often characterised as a Th2-skewed immune response, this is likely to be an oversimplification. The mechanisms underlying innate and adaptive immune dysfunction in food allergy require deeper investigations. At the clinical level, questions about the efficacy and safety of vitamin D in preventing food allergy will only be answered by randomized controlled trials. 
